# The Importance of G Protein-Coupled Receptor Kinase 4 (GRK4) in Pathogenesis of Salt Sensitivity, Salt Sensitive Hypertension and Response to Antihypertensive Treatment

**DOI:** 10.3390/ijms16035741

**Published:** 2015-03-12

**Authors:** Brian Rayner, Raj Ramesar

**Affiliations:** 1 Division of Nephrology and Hypertension, University of Cape Town, Cape Town 7925, South Africa; 2 MRC Human Genetics Research Unit, Division of Human Genetics, Institute of Infectious Diseases and Molecular Medicine, Faculty of Health Sciences, University of Cape Town, Cape Town 7925, South Africa; E-Mail: raj.ramesar@uct.ac.za

**Keywords:** salt sensitivity, *GRK4*, ethnicity

## Abstract

Salt sensitivity is probably caused by either a hereditary or acquired defect of salt excretion by the kidney, and it is reasonable to consider that this is the basis for differences in hypertension between black and white people. Dopamine acts in an autocrine/paracrine fashion to promote natriuresis in the proximal tubule and thick ascending loop of Henle. G-protein receptor kinases (or GRKs) are serine and threonine kinases that phosphorylate G protein-coupled receptors in response to agonist stimulation and uncouple the dopamine receptor from its G protein. This results in a desensitisation process that protects the cell from repeated agonist exposure. *GRK4* activity is increased in spontaneously hypertensive rats, and infusion of *GRK4* antisense oligonucleotides attenuates the increase in blood pressure (BP). This functional defect is replicated in the proximal tubule by expression of *GRK4* variants namely p.Arg65Leu, p.Ala142Val and p.Val486Ala, in cell lines, with the p.Ala142Val showing the most activity. In humans, *GRK4* polymorphisms were shown to be associated with essential hypertension in Australia, BP regulation in young adults, low renin hypertension in Japan and impaired stress-induced Na excretion in normotensive black men. In South Africa, *GRK4* polymorphisms are more common in people of African descent, associated with impaired Na excretion in normotensive African people, and predict blood pressure response to Na restriction in African patients with mild to moderate essential hypertension. The therapeutic importance of the *GRK4* single nucleotide polymorphisms (SNPs) was emphasised in the African American Study of Kidney Disease (AASK) where African-Americans with hypertensive nephrosclerosis were randomised to receive amlodipine, ramipril or metoprolol. Men with the p.Ala142Val genotype were less likely to respond to metoprolol, especially if they also had the p.Arg65Leu variant. Furthermore, in the analysis of response to treatment in two major hypertension studies, the 65Leu/142Val heterozygote predicted a significantly decreased response to atenolol treatment, and the 65Leu/142Val heterozygote and 486Val homozygote were associated in an additive fashion with adverse cardiovascular outcomes, independent of BP. In conclusion, there is considerable evidence that *GRK4* variants are linked to impaired Na excretion, hypertension in animal models and humans, therapeutic response to dietary Na restriction and response to antihypertensive drugs. It may also underlie the difference in hypertension between different geographically derived population groups, and form a basis for pharmacogenomic approaches to treatment of hypertension.

## 1. Introduction

Hypertension is a complex, poorly understood disorder with strong environmental and genetic influences. Salt sensitivity is a term designated to define a group of individuals with a greater rise in blood pressure (BP) after salt loading or a greater fall in blood pressure after salt restriction [1]. People of African descent (hereafter referred to as Blacks) appear to be more salt sensitive than people of Caucasian/European origins (hereafter referred to as Whites) [2]. Greater sodium retention is thought to underlie the BP-determining physiology of Blacks, but specific mechanisms have not been identified. Additionally, African Americans have a higher prevalence of hypertension and morbidity from hypertension for comparable levels of BP, when compared to Whites [3,4].

In a recent prospective observational study in the United States, involving children and adults, plasma renin activity (PRA) and plasma aldosterone concentration were significantly lower in Blacks compared to Whites, and there was a greater rise in 24 h ambulatory BP, brain natriuretic peptide and body weight after treatment with 9-α fludrocortisone indicating greater sodium (Na) retention by the kidney [5]. Similarly, Rayner *et al*. [6] showed that PRA and plasma aldosterone concentrations were significantly lower in South African Blacks compared to Whites for comparable intake of Na, possibly suggesting an underlying genetic predisposition to Na retention.

Regulation of Na balance is critical for the survival of land-based animals. Every day the kidney filters approximately 25,000 mmol of Na, with 65% reabsorbed in the proximal tubule, 27% in ascending loop of Henle, 5% in the distal convoluted tubule and 3% in the collecting duct [7]. As dietary intake ranges from as little as 20 mmols/day (ultra-low, hunter-gatherer diet) to as high as 300 mmols/day (in a typical Western diet), it is essential for the kidney to maintain Na homeostasis by reabsorbing over 99% of the filtered Na. Any defect in Na reabsorption (such as occurs in Barter’s syndrome) results in Na wasting, hypotension, and massive activation of the renin-angiotensin-aldosterone system, often incompatible with long term survival [8]. Barter’s syndrome is due to a “loss of function” mutation in the Na-K-2Cl transporter located in the thick ascending loop of Henle [8]. Conversely, increased function of Na transporters in the kidney, e.g., the epithelial Na channel, results in Liddle’s syndrome characterised by hypertension, hypokalaemia, and suppressed PRA and aldosterone [8].

Bochud *et al.* [9] showed that fractional excretion of Na in the proximal convoluted tubule is highly hereditable, and fractional excretion of Na was significantly higher in Black South Africans when compared to White Belgians. Dopamine is an important regulator of Na balance in the proximal convoluted tubule, and *GRK4* is a serine and threonine kinase that phosphorylates G protein-coupled receptors in response to agonist stimulation. It uncouples the dopamine receptor from its G protein. In this review, the importance of GRK-4 single nucleotide polymorphisms (SNPs) in determining salt sensitivity and salt sensitive hypertension response to dietary and antihypertensive therapy in people of African descent will be explored.

## 2. Dopamine, G-Protein-Coupled Receptors and *GRK4*

**Figure 1:**
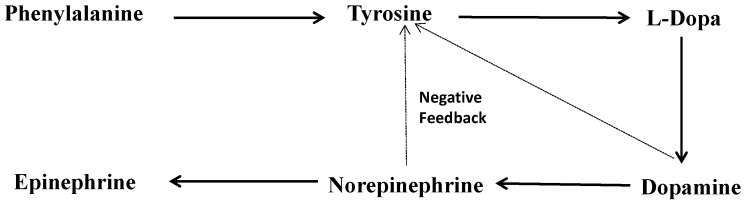
Biosynthesis of catecholamines.

The catecholamines, dopamine and norepinephrine are synthesized from the same precursors; namely, the amino acid tyrosine and its hydroxylated product L-DOPA. (Figure 1) In the kidney, dopamine is mainly synthesised in the proximal tubules by decarboxylation of L-DOPA, which is transported to the tubules from the circulation as the renal tubules are unable to synthesise L-DOPA due to lack of tyrosine hydroxylase. The proximal tubules also lack dopamine β-hydroxylase, and there is no conversion to norepinephrine. Dopamine receptors are present on both the basolateral and apical membranes [10].

Dopamine secreted by the tubules acts in an autocrine/paracrine fashion to regulate Na transport mainly in the renal proximal tubule and thick ascending loop of Henle [10]. The dopamine receptors are distributed widely in the kidney, and the D_1_ receptors (D_1_R) belong to a super family of G protein-coupled receptors, which stimulate adenyl cyclase and protein kinase A. Under conditions of Na excess, locally generated dopamine acts on D_1_R of the renal tubular cells to reduce Na transport and promote natriuresis. Dopamine inhibits renal Na transport by inhibiting the Na-H exchanger, and Na/phosphate co-transporter in apical membranes, and Na/HCO_3_^−^ co-transporter and Na K-ATPase in basolateral membranes in the proximal tubule and thick ascending loop of Henle. Renal dopamine is responsible for over 50% of the incremental Na excretion in response to increased intake [11].

**Figure 2:**
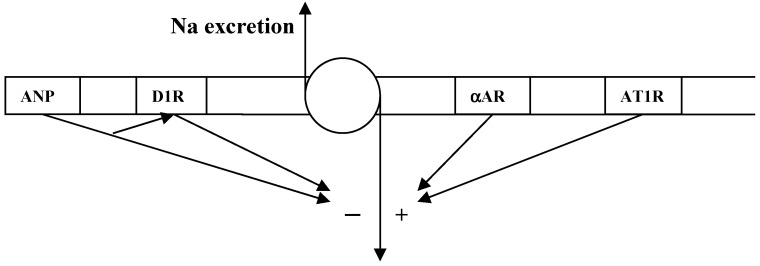
Bidirectional regulation of proximal tubular Na [10]. ANP = atrial natriuretic peptide receptor, D_1_R = dopamine receptor, αAR = α adrenergic receptor, AT1R = angiotensin 1 receptor.

Dopamine interacts with atrial natriuretic peptide (ANP), angiotensin II and α adrenergic receptors in the proximal tubule to maintain Na homeostasis [11]. The model for bidirectional regulation of tubular Na transport is shown in Figure 2. There is evidence that dopamine opposes the anti-natriuretic effect of ANG II, both in the short and long term. It stimulates the activity of proximal tubular Na/K ATPase, which is abolished by dopamine or its messenger cAMP [11]. In fish, dopamine is the predominant catecholamine, but in the terrestrial environment, where Na is a relatively scarce resource, norepinephrine predominates and is essential in the regulation of the anti-natriuretic forces by stimulating the Na/K ATPase in the proximal tubule. Dopamine opposes the action of norepinephrine. Atrial natriuretic peptide (ANP) is an important natriuretic hormone but it requires the presence of dopamine receptors for its full effect. The inhibitory effect of dopamine on the Na/K ATPase is potentiated by ANP.

The effects of dopamine in the kidney provide a physiological model for salt-sensitive hypertension. Any defect in the dopaminergic system will result in unopposed action of ANG II and norepinephrine, and impaired action of ANP on the proximal tubule, resulting in a pronounced antinatriuretic effect [11].

Current research suggests that a defect in the D_1_R is not a likely candidate for salt-sensitive hypertension in humans. However, the uncoupling of the D_1_R from its G protein effector complex is similar to a desensitisation process [12]. This is a mechanism which protects the cell from repeated agonist exposure. Desensitisation involves several processes including phosphorylation, sequestration and degradation of receptor protein. The initial step in this process (phosphorylation) is mediated by a member of the G protein-coupled receptor kinase (GRK). GRKs are serine and threonine kinases that phosphorylate G protein-coupled receptors in response to agonist stimulation [12]. The phosphorylation of G protein-coupled receptor kinases, including D_1_R, leads to binding with members of the arrestin family of proteins that uncouple the receptor from its G protein, and reduction in the functional response [12].

## 3. The Role of *GRK4* in the Pathogenesis of Salt Sensitivity in Animal Models

In hypertension, there appears to be constitutive desensitisation of the renal D_1_R [11]. This appears to be caused by GRK as decreasing its activity or expression in the proximal tubular cells in hypertensives normalises the ability of D_1_R to increase cAMP. In the proximal tubule, *GRK4* is the most important component, compared to other GRKs. *GRK4* activity is increased in spontaneously hypertensive rats, and infusion of *GRK4* antisense oligonucleotides attenuates the increase in BP [13]. This functional defect is replicated in the proximal tubule by expression of *GRK4* variants in cell lines, and this is rectified by prevention of *GRK4* expression [14]. These three tested variants were the p.Arg65Leu, p.Ala142Val, and p.Val486Ala, with the p.Ala142Val showing the most activity [14]. This impairment of dopaminergic function, as evidenced by the *GRK4* variants in animal models, would allow the antinatriuretic function of ANG II and norepinephrine to be unopposed, which may then be responsible for salt sensitivity and rise in BP. It is therefore an attractive candidate gene for human salt sensitivity and hypertension.

In addition, *GRK4* polymorphisms may affect BP through enhanced activity of AT1 receptors. *GRK4* is expressed in vascular smooth muscle cells of the aorta and heterologous expression of the *GRK4γ* variant p.Ala142Val in A10 cells increased AT1 receptor protein expression and increase in intracellular calcium concentration [15]. Angiotensin II-mediated vasoconstriction of the aorta was also higher in *GRK4γ* p.Ala142Val than in wild-type transgenic mice.

## 4. The Role of *GRK4* Variants on Na Excretion in Humans

Several lines of evidence point to a role for *GRK4* variants in the pathogenesis of salt sensitivity and salt sensitive hypertension in humans, especially in African populations. The p.Arg65Leu allele was also associated with impaired stress-induced Na excretion in normotensive Black men [16]. In a study from South Africa, salt excretion was examined in healthy normotensive and lean black and white men after an acute saline load [17]. The *GRK4* p.Ala142Val polymorphism, which was significantly more frequent in black men, was associated with significantly lower plasma aldosterone concentrations and impaired incremental urinary Na excretion. Hypertension-related polymorphisms and cardiovascular indices were also analyzed in 97 normotensive, healthy Japanese adults. NT-proBNP levels were significantly higher in subjects with two or more *GRK4* polymorphic alleles strongly suggesting enhanced Na retention [18]. Sodium excretion was inversely related to the number of *GRK4* variants in hypertensive persons, and the natriuretic response to dopaminergic stimulation was impaired in normotensive persons having *GRK4* gene variants [19].

## 5. *GRK4* Variants and Hypertension

In an Australian study, involving 168 unrelated white subjects/patients and 312 normotensive controls, *GRK4* polymorphisms were associated with essential hypertension [20]. Furthermore, a recent study linked the *GRK4* variants, in particular p.Arg65Leu, with BP regulation in adolescents and young adults [21]. A Japanese study of hypertensive subjects developed a multi-variant genetic model based on the p.Arg65Leu, p.Ala142Val, and p.Val486Ala changes in *GRK4* which was 94.4% predictive of salt-sensitive hypertension, while a single-variant model with a single p.Ala142Val variant (in *GRK4*) was only 78.4% predictive [19].

**Figure 3:**
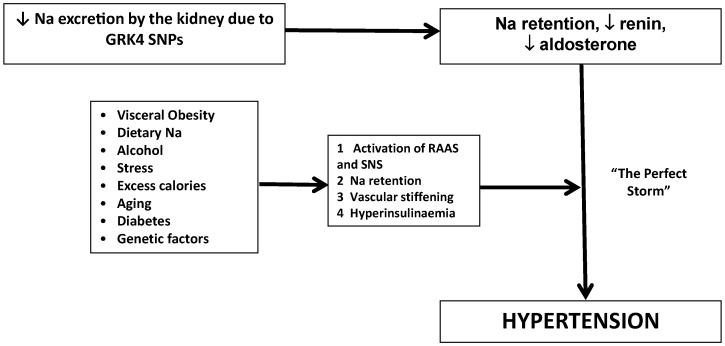
Putative pathogenesis of salt sensitive hypertension related to GRK-4 SNPs. SNS = sympathetic nervous system, RAAS = renin-angiotensin-aldosterone system.

The putative pathogenesis of salt sensitive hypertension due to *GRK4* SNPs is shown in Figure 3 demonstrating the interaction of genes and environment. Environmental factors interplay with the underlying genetic factors to develop the “perfect storm” for the development of hypertension.

## 6. *GRK4* Variants and Response to Dietary and Pharmacological Intervention

In South Africa, *GRK4* polymorphisms are more common in people of African descent, are associated with impaired Na excretion in normotensive people, and predict blood pressure response to Na restriction in African patients with mild to moderate essential hypertension [22]. The therapeutic importance of the *GRK4* SNPs was emphasised in the African American Study of Kidney Disease (AASK) where African-Americans with hypertensive nephrosclerosis were randomised to receive amlodipine, ramipril or metoprolol [23]. Men with the p.142Ala genotype were less likely to respond to metoprolol, especially if they had co-inherited the p.65Leu variant. Furthermore, in the analysis of response to treatment in two major hypertension studies, the 65Leu/142Val haplotype predicted a significantly decreased response to atenolol treatment, and the 65Leu/142Val heterozygote and the 486Val/486Val homozygote were associated in an additive fashion with adverse CV outcomes independent of BP [24]. The effects of GRK-4 variants are summarized in Table 1.

**Table 1:** Summary of the associations of GRK variants with Na excretion and hypertension.

GRK Variant	Parameter	Comment
p.Arg65Leu, p.Ala142Val, and p.Val486Ala	Hypertension in rats	p.Ala142Val shows greatest activity
p.Ala142Val	Enhanced activity of AT1 in smooth muscle	–
p.Arg65Leu	Impaired stress related Na excretion in normotensive blacks	–
p.Ala142Val	Impaired incremental Na excretion in healthy men, and lower aldosterone levels	p.Ala142Val significantly more common in Blacks compared to Whites
p.Arg65Leu, p.Ala142Val, and p.Val486Ala	Impaired Na excretion and higher BNP levels in healthy Japanese with two or more variants	–
p.Val486Ala	Association with hypertension in Australian population	–
p.Arg65Leu, p.Ala142Val, and p.Val486Ala	94.4% predictive of salt sensitive renin hypertension in Japanese population	p.Ala142Val 78.4% predictive
p.Arg65Leu, p.Ala142Val	BP response to Na restriction in black South Africans with hypertension	–
p.142Ala genotype	In AASK males less likely to respond to metoprolol	Especially if co-inherited the p.65Leu variant
65Leu/142Val haplotype	In two major hypertension studies predicted a reduced response to atenolol and increased cardiovascular outcomes	–

## 7. Conclusions

There is now mounting evidence that *GRK4* variants are linked to impaired Na excretion, hypertension in animal models and humans, therapeutic response to dietary Na restriction and response to antihypertensive drugs. It may also underlie the difference in hypertension between individuals of African and European origin, and form a basis to a pharmacogenomics approach to the understanding and treatment of hypertension.
